# CD30-Positive Extracellular Vesicles Enable the Targeting of CD30-Negative DLBCL Cells by the CD30 Antibody-Drug Conjugate Brentuximab Vedotin

**DOI:** 10.3389/fcell.2021.698503

**Published:** 2021-07-30

**Authors:** Liudmila Lobastova, Marcus Lettau, Felix Babatz, Thais Dolzany de Oliveira, Phuong-Hien Nguyen, Bianca Alves Pauletti, Astrid C. Schauss, Horst Dürkop, Ottmar Janssen, Adriana F. Paes Leme, Michael Hallek, Hinrich P. Hansen

**Affiliations:** ^1^Department I of Internal Medicine, University of Cologne, Cologne, Germany; ^2^Center for Integrated Oncology Aachen Bonn Cologne Duesseldorf, Cologne, Germany; ^3^Center for Molecular Medicine Cologne, University of Cologne, Cologne, Germany; ^4^CECAD Center of Excellence on Cellular Stress Responses in Aging-Associated Diseases, Cologne, Germany; ^5^Institute of Immunology, Christian-Albrechts-University of Kiel and University Hospital Schleswig-Holstein, Kiel, Germany; ^6^Department of Hematology, University Hospital Schleswig-Holstein, Kiel, Germany; ^7^CECAD Center of Excellence on Cellular Stress Responses in Aging-Associated Diseases, Imaging Facility, Cologne, Germany; ^8^Laboratório de Espectrometria de Massas, Laboratório Nacional de Biociências, Centro Nacional de Pesquisa em Energia e Materiais, Campinas, Brazil; ^9^Pathodiagnostik Berlin MVZ GmbH Berlin, Berlin, Germany

**Keywords:** tumor microenvironment, cellular crosstalk, immune therapy, antibody-drug conjugate, extracellular vesicle

## Abstract

CD30, a member of the TNF receptor superfamily, is selectively expressed on a subset of activated lymphocytes and on malignant cells of certain lymphomas, such as classical Hodgkin Lymphoma (cHL), where it activates critical bystander cells in the tumor microenvironment. Therefore, it is not surprising that the CD30 antibody-drug conjugate Brentuximab Vedotin (BV) represents a powerful, FDA-approved treatment option for CD30^+^ hematological malignancies. However, BV also exerts a strong anti-cancer efficacy in many cases of diffuse large B cell lymphoma (DLBCL) with poor CD30 expression, even when lacking detectable CD30^+^ tumor cells. The mechanism remains enigmatic. Because CD30 is released on extracellular vesicles (EVs) from both, malignant and activated lymphocytes, we studied whether EV-associated CD30 might end up in CD30^–^ tumor cells to provide binding sites for BV. Notably, CD30^+^ EVs bind to various DLBCL cell lines as well as to the FITC-labeled variant of the antibody-drug conjugate BV, thus potentially conferring the BV binding also to CD30^–^ cells. Confocal microscopy and imaging cytometry studies revealed that BV binding and uptake depend on CD30^+^ EVs. Since BV is only toxic toward CD30^–^ DLBCL cells when CD30^+^ EVs support its uptake, we conclude that EVs not only communicate within the tumor microenvironment but also influence cancer treatment. Ultimately, the CD30-based BV not only targets CD30^+^ tumor cell but also CD30^–^ DLBCL cells in the presence of CD30^+^ EVs. Our study thus provides a feasible explanation for the clinical impact of BV in CD30^–^ DLBCL and warrants confirming studies in animal models.

## Introduction

CD30 is a transmembrane protein of the TNF receptor superfamily (TNFRSF8). It is restricted to a small population of activated T and B immunoblasts in lymphoid tissue of healthy individuals (Stein et al., 1985; Horie and Watanabe, 1998). In T cells, CD30 serves as a costimulatory receptor, which is upregulated by T cell receptor (TCR) and CD28 ligation in addition to the action of cytokines, such as IL-4 (Gilfillan et al., 1998). Normal CD30^+^ B cells are mostly class-switched and express CD27 (TNFRSF7) (Weniger et al., 2018). The corresponding ligand (CD30L/TNFSF8) is also a transmembrane protein, which is weakly expressed on various immune cells including neutrophils, eosinophils, lymphocytes, myeloid cells, and mast cells. Here, the binding of membrane-associated CD30 causes reverse signaling and context-dependent pleiotropic effects, ranging from degranulation-free activation of myeloid cells to B cell inhibition (Wiley et al., 1996; Cerutti et al., 2000). In mice, constitutively active CD30 in B cells is associated with augmented B1-B and plasma cell counts, implicating NF-κB signaling and STAT 3 and STAT6 phosphorylation (Sperling et al., 2019). When aged, such mice have an enhanced risk of lymphomagenesis. Additionally, CD30 is expressed in transformed cells of certain malignant lymphomas, such as classical Hodgkin Lymphoma (cHL) and systemic Anaplastic Large Cell Lymphoma (sALCL; Horie and Watanabe, 1998). Because of its selective expression in malignant cells, CD30 became of interest for targeted immunotherapy with the monomethyl auristatin E (MMAE)-coupled humanized CD30 antibody drug conjugate (ADC) Brentuximab Vedotin (BV; Wahl et al., 2002). After internalization of the ADC, the acidic milieu of the lysosomes releases MMAE that is able to inhibit microtubule formation (Sutherland et al., 2006). In a pivotal study, BV treatment of sALCL and cHL patients, who relapsed after standard therapy, resulted in an overall response rate of 86 and 75%, respectively (Younes et al., 2012). Interestingly, BV proved also effective against other malignancies with less pronounced CD30 expression.

Recent clinical studies revealed that a minor percentage of diffuse large B cell lymphoma (DLBCL) patients had tumor cells with CD30 expression (Hu et al., 2013). Visually assessed immunohistochemistry (IHC) identified CD30^+^ tumor cells in about 25% of DLBCL cases (Slack et al., 2014). However, unbiased computer-assisted IHC, which calculates also non-malignant CD30^+^ cells, identified higher case numbers (36%) (Bartlett et al., 2017). Remarkably, clinical trials with BV in DLBCL patients suggested that the response to BV-treatment did not correlate with the amount of CD30^+^ tumor cells. In one case, a complete remission was achieved with BV even when CD30^+^ tumor cells were not detectable (Jacobsen et al., 2015). These data suggest that the CD30 transfer from non-malignant cells might contribute to the clinical efficacy of BV.

Extracellular vesicles (EVs) are released by virtually all cell types. They originate from the outward budding of multivesicular endosomes or budding from the plasma membrane. Endosome-derived EVs are referred to as small EVs (s-EVs, exosomes) and are generally smaller than plasma membrane-derived large EVs (l-EVs or microvesicles) (Thery et al., 2009; Nishimura et al., 2021). Both EV types are membrane-enclosed particles and harbor typical traits of the donor cells, including nucleic acids, lipids, and soluble and membrane-associated proteins. By binding to or fusing with cells in the proximity or at distant sites, EVs are able to communicate *in trans* in a cell contact-like manner with recipient cells and thus undoubtedly represent an important intercellular communication tool (Tkach and Thery, 2016).

In a previous study, we showed that EVs of cHL cells, carry CD30 as a cargo and bind to and are taken up by typical bystander cells (Hansen et al., 2016). In the presence of BV, the CD30^+^ EVs are able to mediate cell damage also to CD30^–^ cells in a CD30^+^ EV-dependent manner. Thus, the transport of CD30 to bystander cells enables a dual targeting of CD30^+^ tumor and CD30^–^ supporter cells. Since DLBCL presents as a heterogeneous disease, encompassing cases with variable CD30^+^ tumor and/or CD30^+^ bystander cells, a functional EV-dependent transport from CD30^+^ cells to CD30^–^ DLBCL cells might explain the efficacy of BV in cases with CD30^–^ tumor cells. In our study, we provide strong evidence, that CD30^+^ EVs are able to bind and transport BV functionally to CD30^+^ or CD30^–^ DLBCL cells.

## Materials and Methods

### Cells and Reagents

The cell lines L540, THP-1, P30/OHKUBO and DoHH-2 were purchased from the DSMZ (Braunschweig, Germany). Karpas 422 was from Sigma Aldrich (St. Louis, MO, United States). Cells were cultivated at 37°C and 5% CO_2_ in RPMI 1640 containing 10% FBS, supplemented with GlutaMAX, 100 U/mL penicillin and 100 μg/mL streptomycin. The following additional reagents were used: PE Annexin V (RRID: AB_2561298), PE anti-human CD30 (RRID: AB:2207595), PE goat anti mouse IgG (RRID: AB_315010), anti-human CD81 (RRID: AB_10643417), anti-human CD9 (RRID: AB_314907), anti-human CD63 (RRID: AB_11204263), anti-human CD54 (RRID: AB_535974). The SGN30 antibody was from Seattle Genetics (Bothell, WA, United States). In fluorescence studies, the antibody was coupled with FITC (Sigma) in a 0.2 M carbonate buffer at pH 9.2 and purified by subsequent gel filtration. The CD30 antibody Ki-3 (Horn-Lohrens et al., 1995) was labeled with the deep red fluorescence dye CF 594 (Sigma) according to the manufacturer’s instruction. The sheddase inhibitor Ro 32-7315 was kindly provided by Roche Diagnostics, Penzberg, Germany. The anti-CD30 hybridoma HeFi-1 was kindly provided by Dr. Ellis, Loyola Univ., Maywood, IL, United States.

### Vesicle Isolation

Cells were cultivated under serum-free conditions at 5 × 10^6^/mL for 2 h in the presence of 10 μM Ro 32-7315. Cell supernatant was collected and cleared by three consecutive centrifugation steps, i.e., 5 min at 300 × *g*, 10 min at 1,000 × *g*, 20 min at 3,500 × *g*. Cleared supernatants were sedimented in an ultracentrifuge for 2 h at 100,000 × *g*. Vesicle-containing pellets were washed twice with PBS by ultracentrifugation. Particle concentration and size distribution of isolated EVs were determined by nanoparticle tracking analysis (NTA, Nanosight NS300, Malvern Instruments). The means of five consecutive measurements of 1 min were evaluated to adjust the EV concentration to 1 × 10^10^ EVs/mL.

### Flow Cytometry of EVs

Extracellular vesicles were incubated at 4°C overnight with 4.5 μm Polybead carboxylate microspheres (Polysciences, Warrington, PA, United States). The beads were blocked with 1% BSA (v/w) in PBS for 1h at 800 rpm. Then, aliquots were incubated with fluorescence-labeled antibodies. Beads were evaluated by flow cytometry on a Gallios device (Beckman Coulter).

### Electron Microscopy

Extracellular vesicles were fixed with 2% formaldehyde (Merck). A 100 mesh formvar coated copper grid (Science Services) was placed onto 5 μL of sample and incubated for 20 min. Grids were washed seven times for 2 min in PBS and place for 5 min on a drop of 1% glutaraldehyde (Merck). Grids were washed eight times in ddH_2_O. Grids were placed for 4 min onto a drop of 1.5% aqueous uranyl acetate and blotted with a filter paper. Images were acquired using a JEM-2100 Plus Transmission Electron Microscope (JEOL) operating at 80kV equipped with a OneView 4K camera (Gatan).

### EV-Labeling With DiD

Extracellular vesicles were isolated and washed with PBS by ultracentrifugation at 100,000 × *g* and subsequently labeled with 40 nM of the membrane dye DiD (1,1′-Dioctadecyl-3,3,3′,3′- tetramethylindo-dicarbo-cyanine, 4-Chlorobenzenesulfonate salt, Thermo Fisher Scientific). Then, EVs were incubated for 20 min at 37°C and washed by ultracentrifugation at 100,000 × *g* for 1 h at 4°C. Afterward DiD-labeled EVs were added to the target cell lines, incubated for 2 h at 4°C and cells were then analyzed by flow cytometry.

### LC-MS/MS

The peptide mixture was analyzed in an LTQ Orbitrap Velos (Thermo Fisher Scientific) mass spectrometer coupled to nanoflow liquid chromatography on an EASY-nLC system (Proxeon Biosystems) with a Proxeon nanoelectrospray ion source. The parameters for MS analysis are described in further detail in Supplementary Figure 1. The mass spectrometry proteomics data have been deposited to the ProteomeXchange Consortium via the PRIDE partner repository with the dataset identifier PXD025442 (Perez-Riverol et al., 2019).

### Confocal Laser-Scanning Microscopy

Eight-well μ-slides (Ibidi) were coated over night with 0.01% poly-L-lysine at 37°C and afterwards washed three times with medium. Cells were let to adhere and incubated with Ki-3-CF594-loaded EVs for 1 h. Then, the medium was carefully removed and replaced with fresh medium, containing CellMask^TM^ Deep Red Plasma membrane stain (649/666 nm; Thermo Fisher Scientific) and Hoechst 33342 dye (NucBlue^TM^ Live ReadyProbes^TM,^ Thermo Fisher Scientific). After 5 min incubation, the dye-containing medium was substituted by fresh medium and the samples immediately subjected to the confocal laser scanning microscope (Leica TCS SP8, 63x PlanApo oil objective N.A. 1.4) with super resolution (∼50 nm lateral, 120 nm axial).

### Measurement of EV Uptake by Imaging Flow Cytometry (ImageStream)

Target cells (5 × 10^5^) were washed twice with FACS buffer before incubation with SGN30-FITC ± EVs for 1 h at RT to allow EV binding and internalization. Then, cells were washed once with cold FACS-buffer and then fixed with 1% PFA in PBS and subsequently kept on ice until internalization could be analyzed by imaging flow cytometry.

Internalization of FITC-labeled SGN30 antibody and CD30^+^ EVs by Karpas 422 or P30-OH/KUBO cells was quantified by imaging flow cytometry using an ImageStream X Mark II one camera system with 351, 488, 562, 658 and 732 nm lasers (Merck Millipore, Burlington, MA, United States). The system was calibrated using SpeedBeads (Merck Millipore) prior to use and at least 10,000 events with an area >12.5 μm^2^ based on bright field images were acquired. Moreover, 500–1,000 events of single stained compensation control samples gated on appropriate signal size were acquired with both the bright field channel and the 732 nm laser turned off. Images (bright field in channel 1 and FITC in channel 2 (505–560 nm) were acquired at 60-fold magnification. The integrated software INSPIRE^®^ was used for data collection as raw image files. Single color controls were used to calculate a spectral crosstalk matrix that was applied to each raw image file for spectral compensations in the detection channels. The analysis was performed on the compensated image files using the IDEAS^®^ image analysis software. The bright field gradient root mean square (RMS) feature was used to gate on focused cells and dot plots of the bright field area versus the aspect ratio were used to gate on single cells. The internalization wizard was used to calculate the internalization score that is defined as the ratio of the intensity inside the cell to the intensity of the entire cell mapped to a logarithmic scale.

### Toxicity Test

Target cells (1 × 10^5^ cells/well) were cultivated in 500 μL medium/well in a 24-well plate ± 10^9^ EVs from L540 or THP-1 along with different amounts of BV ± HeFi-1 antibody (1 μg/mL final concentration) or medium alone as control. After 24 h another 250 μL of culture medium was added to each well. The cells were further incubated for 48 h and cell viability was assessed by flow cytometry. To this end, the cells were washed once with FACS buffer, then stained with propidium iodide (PI; 0.5 μg/mL) for 30 min at 4°C, washed twice with FACS buffer and PI^+^ cells were determined by flow cytometry.

### Statistical Analysis

If not stated otherwise, all experiments were performed in at least three independent replicates. Results obtained from representative experiments are shown. Data (*n* ≥ 3) are presented as mean ± standard errors of the mean (SEM) and were analyzed using GraphPad Prism 7 software. Statistical significance was calculated as indicated in the Figures.

## Results

### Production of CD30^+^ EVs

**FIGURE 1:**
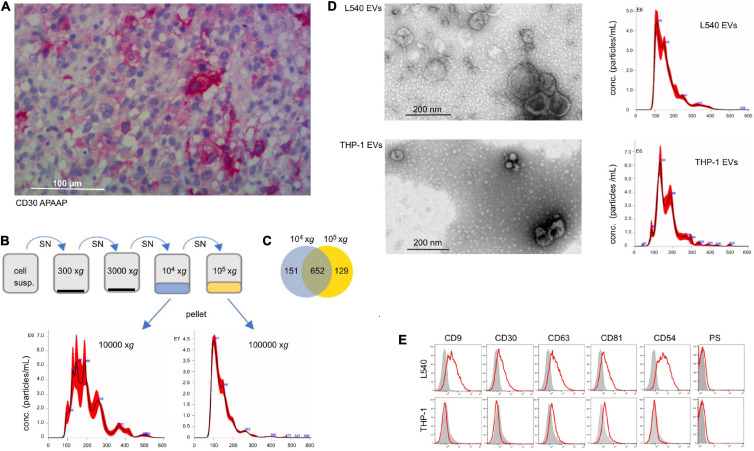
Characterization of CD30^+^ EVs. **(A)** Tissue section of lymph node infiltrated with DLBCL cells, which was stained with CD30 mAb (Ber-H2). The bar indicates 100 μm. **(B)** Characterization of EVs from the cell supernatant of L540 cells. They were isolated by a series of centrifugation steps. The 10,000 × *g* and the subsequent 100,000 × *g* fraction were studied by NTA and mass spectrometry. **(C)** The common and individual mass spectrum hits are shown in a Venn diagram. **(D)** The pooled 10,000 × *g* and 100,000 × *g* EV fractions of L540 and THP-1 supernatants were studied by transmission electron microscopy. The diameter (x-axis) and count (y-axis) of the EVs from the cell supernatant of L540 and THP-1 cells were analyzed by NTA. **(E)** The EVs were bound to 10 μm microspheres and analyzed by flow cytometry. The EVs were stained for CD9, CD30, CD63, CD81, CD54, and phosphatidylserine (red) and compared to the isotype controls (gray).

In many cases of DLBCL, cells with variable expression of CD30 are admixed in the affected tissue. They are either malignant themselves or reactive immunoblasts (Figure 1A). Here, we studied whether EVs from CD30^+^ cells contribute to the efficacy of BV in DLBCL. In order to generate CD30^+^ EVs for subsequent experiments, we cultivated the CD30^+^ L540 cell line for 1 h under serum-free conditions and harvested the cell supernatant. The l-EVs sediment at lower gravity than the s-EVs (Nishimura et al., 2021). In order to determine, which EV type preferentially contains CD30, we compared EVs that sediment at different gravity. After two centrifugation steps at low gravity to remove cell debris, we collected vesicles that sediment at 10,000 × *g* and another portion from a subsequent ultracentrifugation step at 100,000 × *g* (Figure 1B). Nanoparticle tracking analysis (NTA) revealed that the 10,000 × *g* fraction contained roughly 4× less but larger vesicles than the 100,000 × *g* fraction, i.e., 6.97 × 10^8^ ± 3.94 × 10^7^ particles/mL with a mean diameter of 205 nm and 3.24 × 10^9^ ± 1.03 × 10^8^ with a mean diameter of 143 nm, respectively. Because both particle fractions are deliberately released into the environment, they are candidate vehicles, provided they contain CD30. Mass spectrometry demonstrated that both EV fractions yielded a similar amount of protein hits with largely overlapping genes, including CD30, i.e., 81% of the 10,000 × *g* and 83% of the 100,000 × *g* fraction (Figure 1C and Supplementary Table 1). Interestingly, both EV preparations contained almost exclusively hits, which are found in the EV protein database Vesiclepedia, i.e., 98.5 and 97.3% in the 10,000 × *g* and 100,000 × *g* fractions, respectively (Supplementary Figure 1). Also, categorization of the protein hits with the Funrich 3.1.4 software revealed that similar percentages were allocated to different cellular components, whereof the category of exosomes was the most prominent one. Because of the similar protein composition, particularly the presence of CD30, we pooled the 10,000 × *g* and the 100,000 × *g* fraction for further experiments. EVs from the CD30^–^ cell line THP-1 were tested as a control. Transmission electron microscopy of the pooled fractions identified in both cases (OsO_4_-stained) membrane-enclosed particles of variable diameter within the range of EVs. NTA measurement showed a similar EV diameter in the pooled fractions from L540 and the THP1 cell, i.e., 165.0 ± 67.5 nm (L540) and 172 ± 63.9 nm (THP-1) (Figure 1D). As expected, EVs from both cell types expressed typical EV markers such as phosphatidylserine and tetraspanins (CD63, CD81, and CD82) but only EVs from L540 cells contained CD30 (Figure 1E). Therefore, we used L540 EVs as CD30 carriers and THP-1 EVs as negative controls in subsequent experiments.

### Targeting of Leukemia Cells With CD30^+^ EVs

**FIGURE 2:**
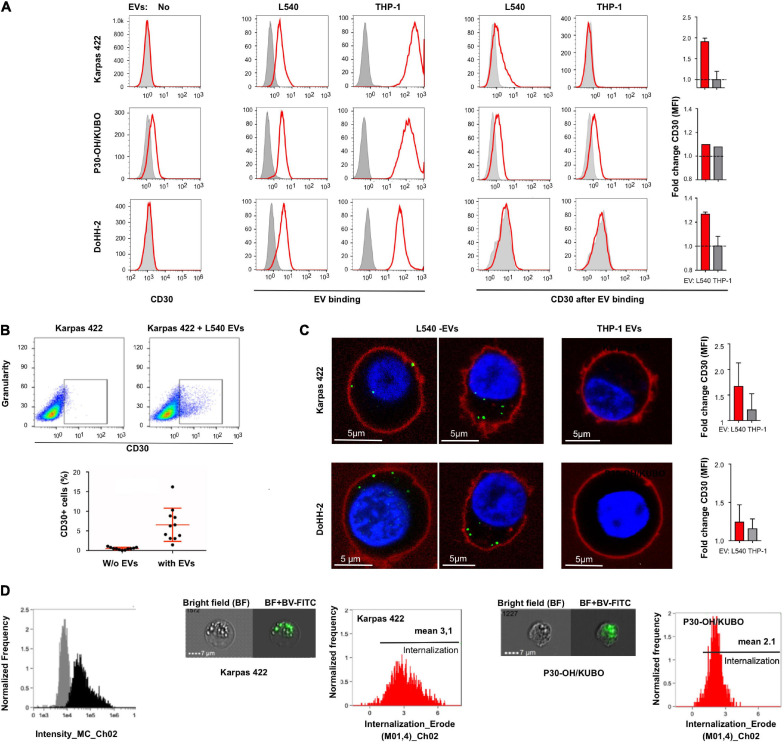
Extracellular vesicle-dependent binding and incorporation of CD30 and BV. **(A)** The first column of graphs shows a flow cytometric analysis of the CD30 expression on the target cells Karpas 422, P30-OH/KUBO and DoHH-2 (red) compared to the isotype control (gray). The next two columns show the binding/uptake of purified EVs from the supernatant of L540 or THP-1 cells (red). For that, the cells were treated for 2 h at 4°C with DiD-labeled EVs. Untreated cells served as controls (gray). To the right, the two columns show the amount of CD30 after binding of EVs from L540 or THP-1 cells (red) as compared to the isotype control (gray). The adjacent bars indicate the relative increase of the CD30 mean fluorescence intensity (MFI) by the indicated EVs. **(B)** CD30 expression of Karpas 422 measured by flow cytometry, first gated on living cells and then the percentage of CD30^+^ cells was calculated with and without EVs; shown as a representative image without EVs **(upper left)** and with EVs **(upper right)**, and in a diagram summarizing the results of 11 measurements. **(C)** Confocal microscopy of Karpas 422 and DoHH-2 with L540 EVs **(left panel)** or THP-1 EVs **(right)**. Cells were incubated with Ki-3-CF594-loaded EVs and the cell membrane was subsequently stained with CellMask deep red. The fold change of CD30 expression after L540 and THP-1 EVs binding was measured by flow cytometry and normalized to the samples without EVs. **(D)** The internalization process was further analyzed by one imaging flow cytometry investigation. The specific intensity of the green fluorescence is depicted [**(left)**, black]. Representative images are shown of Karpas 422 and P30-OH/KUBO cells, incubated with FITC-labeled SGN30 and EVs from L540 cells for 60 min at 37°C. Scale bars represent 7 μm. Histograms displaying the internalization score are shown.

To study the efficacy of CD30^+^ EVs in mediating BV toxicity in non-Hodgkin B cell leukemia, we examined three target cell lines for endogenous CD30 expression, binding of donor cell EVs and enrichment of CD30 through EV binding. Flow cytometry showed that the cell lines Karpas 422 and DoHH-2 (GC-DLBCL) had no detectable CD30, whereas P30-OH/KUBO was positive for CD30 (Figure 2A, left). All tested cell lines were able to bind or take up fluorescence-labeled EVs from both L540 and THP-1 cells (Figure 2A, middle). As expected, only the addition of EVs from L540 cells cumulated CD30 at the cell surface of the CD30^–^ cell lines (Figure 2A, right). We determined the EV-dependent enrichment of CD30 in the cells by flow cytometry, comparing the CD30-depending mean fluorescence intensity between untreated and EV-treated cells. The bars indicate, that among the tested cells, Karpas 422 showed better cell surface enrichment of EV-borne CD30 than DoHH-2 after 2 h of incubation time. In P30-OH/KUBO with endogenous CD30, EVs from L540 did not contribute much to the overall cell surface expression of CD30. Our data showed that universally membrane-labeled EVs generate a stronger signal on target cells than the anti-CD30 staining. As shown in a dot-plot, only a limited percentage of target cells accumulates CD30^+^ EVs (median 0.52–6.55%, *N* = 11; Figure 2B). This is not surprising because EVs from one source are generally very heterogeneous in protein composition and only a certain percentage might contain CD30 (Lee et al., 2018). In addition, the internalization rate of CD30 might be different between cell types upon ligation, and a variable amount of CD30 might have been internalized following antibody binding. Because CD30 antibody internalization is the aim in therapy, we studied the internalization of anti-CD30 antibody. To approach this question, we performed a series of imaging experiments, after coincubation of target cells with EVs and fluorescent anti-CD30 antibody. Unfortunately, the antigen binding of BV dropped after labeling with FITC, so for this set of experiments we used either SGN30, which is the humanized backbone of BV or the murine CD30 antibody Ki-3, which belongs to the same serological cluster as SGN30/35 (Horn-Lohrens et al., 1995). Confocal microscopy indicated that both CD30^–^ Karpas 422 and DoHH-2 have a clear CD30^+^ EV-dependent uptake of CD30 antibody (Figure 2C). CD30^–^ EVs from THP1 cells did not support this effect. Microscopy suggested a stronger signal inside the cells than on the cell surface, partially explaining the low EV-dependent surface CD30 in Figures 2A,B. Quantification of the antibody uptake was approached by imaging flow cytometry (Figure 2D). We determined the internalization score of SGN30-FITC from cells incubated with L540-EVs, resulting in values of 3.1 (*N* = 2156) for Karpas 422 cells and 2.1 (*N* = 1086) for P30-OH/KUBO cells. These data indicate that both cell types display uptake of the BV antibody backbone in the presence of CD30^+^ EVs.

**FIGURE 3:**
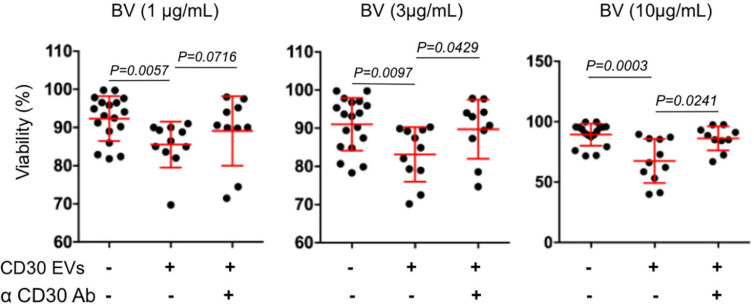
Viability assay of Karpas 422. Viability assay of Karpas 422 ± EVs from L540 cells with the addition of BV in different concentrations 1, 3, and 10 μg/mL (from left to right); ± the inhibitory anti-CD30 antibody HeFi-1. The percentage of living cells was analyzed by flow cytometry using the propidium iodide (PI) staining. For the statistical analysis the Mann–Whitney test was used.

**FIGURE 4:**
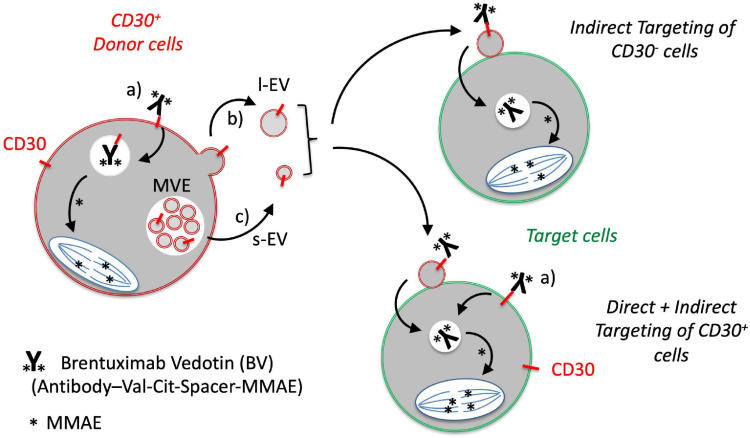
Proposed model for the role of EVs in the targeting of CD30-negative tumor cells with the CD30 antibody drug conjugate BV. CD30 is expressed by a number of malignant DLBCL cells and activated immunoblasts. a) The CD30 ADC BV binds to CD30^+^ tumor cells, is internalized and the cytotoxic compound monomethylauristatin E (MMAE; ^∗^) is selectively cleaved and activated at the dipeptide valine (Val)-citrulline (Cit) by the lysosomal peptidase cathepsin B. CD30 is also released in b) large EVs (l-EVs) by budding from the surface and c) in small EVs (s-EVs) by plasma membrane fusion of multivesicular endosomes. d) CD30^+^ EVs bind BV and target different cells within the tumor microenvironment, such as CD30^+^ or CD30^–^ DLBCL cells. This crossfire effect might be responsible for the strong clinical efficacy of BV even in cases with CD30^–^ tumor cells.

We also determined the functionality of CD30^+^ EVs regarding BV-induced cytotoxicity in CD30^–^ Karpas 422 cells (Figure 3). BV alone had no significant influence on the cell viability after three days of incubation, resulting in 93.6, 93.4, and 92.2% viability after treatment with 1, 3 and 10 μg/mL BV, respectively. However, after the addition of CD30^+^ EVs (1 × 10^9^/mL), the cell viability decreased significantly upon BV treatment, consequently resulting in cell viability of 86.5, 84.9, and 66.2% respectively. This effect was dependent on CD30 because the addition of the competitive CD30 antibody HeFi-1 could neutralize the toxicity, yielding improved cell viability of 90.3, 91.9, and 86.9%. The antibody-dependent neutralization provides a strong argument that the cytotoxicity of BV in such CD30^–^ target cells is indeed dependent on CD30^+^ EVs. We also tested the influence of CD30^+^ EVs on the toxicity of BV in the CD30^+^ cell line P30-OH/KUBO (Supplementary Figure 2). Although these cells constitutively express CD30 and BV is able to directly target them, this experiment provides initial evidence that CD30^+^ EVs might contribute to or enhance the toxicity of BV even in CD30^+^ cells (Figure 4).

## Discussion

The functional efficacy of the CD30 ADC BV is well-established in the treatment of lymphomas with high expression of CD30 in tumor cells (Younes et al., 2012). In DLBCL, the CD30 expression is variable, ranging from cases with a high percentage to cases without detectable CD30 expression. Depending on the sensitivity of the readout and whether bystander cells were included in the calculation, the CD30^+^ cases range from 12 to 36% (Slack et al., 2014). However, DLBCL patients without CD30^+^ tumor cells still profit from the BV therapy, in rare cases even with complete remission. This observation raises the question of the underlying mode of action in CD30^–^ tumor cells. Here, we studied a possible function of CD30^+^ EVs conveying the anti-tumor functionality of BV. Major findings of our study are that (i) the targeting antigen CD30 is present on l-EVs and s-EVs from L540 cells, (ii) such EVs bind to both, the immunotoxin BV and the DLBCL cells, (iii) EVs are taken up by target cells, and (iv) dependent on BV and CD30^+^ EVs, such BV-loaded EVs also damage CD30^–^ DLBCL cells dependent on BV and CD30^+^ EVs (Figure 4).

In most cases, tumor cells do not grow independently but require the support of other malignant cells and tumor-(re)programmed non-malignant bystander cells. Therefore, in addition to addressing the malignant cells quantitatively, the targeting of the supporting tumor microenvironment has become an emerging focus of modern cancer therapy. As an example, the targeting of tumor-supporting CD25^+^ Treg cells with ^90^Y-daclizumab (anti-CD25) significantly improves clinical responses in cHL patients (Janik et al., 2015). This effect has also been explained by the collateral damage of the radio isotope, targeting primarily tumor-rosetting T cells and also neighboring tumor cells. Such a crossfire effect was also suggested for the functionality of BV. After uptake of BV and cleavage of the toxic MMAE in the target cells, it was suggested that a small amount of MMAE passively penetrates the plasma membrane and diffuses back into the cell environment where it damages neighboring cells (Okeley et al., 2010).

In the last decade, the understanding of the role of EVs in the intercellular communication has significantly improved. Such EVs are also advancing cancer treatment and their influence on the PD1/PD-L1 system might be regarded as a typical example. In many malignant tumors, the immune surveillance fails because the malignant cells express programmed cell death ligands such as PD-L1, which, after cell contact, inhibit PD-1-expressing immune surveillance cells. Recently, it was shown, that malignant cells also release PD-L1 on EVs, which increases the extent of immunosuppression (Chen et al., 2018). EVs are small, mobile membrane-enclosed particles, which are released by virtually all cell types. They are regarded as fingerprints of the donor cell and thus carry and present many typical traits of the originating cell to malignant or bystander cells of the microenvironment, in a membrane-associated context. For example, chronic lymphocytic leukemia cells can activate each other during disease progression by transferring S100-A9 in EVs (Prieto et al., 2017).

CD30 is also transported by l-EVs and s-EVs along with its active releasing enzyme ADAM10. Because CD30 is expressed almost exclusively by certain tissue-associated cells and the EVs thereof, the releasing enzyme processed most CD30 before such EVs end up in the circulation (Hansen et al., 2016). This explains how blood contains predominantly the proteolytically processed soluble product sCD30. Our model argues that CD30 is only transiently expressed on EVs and the highest concentration and functionality is restricted to the vicinity of the donor cell. Because (i) CD30 antibodies are poorly internalized in many CD30^+^ cell lines (Matthey et al., 2004), (ii) EVs together with CD30 antibody show a good uptake in confocal microscopy and imaging flow cytometry, and (iii) CD30 EVs contribute to the toxicity of BV even in the CD30^+^ target cell P30-OH/KUBO (Supplementary Figure 2), we speculate that BV-loaded EVs play a major role in the therapeutic functionality of BV. However, animal models might be helpful to further substantiate our findings.

At the moment it is still not clear why CD30 does not accumulate in large quantities at the target cell surface, whereas EVs do. We know from earlier immunoelectron microscopic studies that many EVs from L540 cells do not carry CD30 (Hansen et al., 2014). In particular, very small EVs more often lack CD30 compared to larger ones. This is also a reason not to focus on small EVs alone in this study and also include larger vesicles from the 10,000 × *g* fraction. One might speculate that a certain EV type preferentially participates in EV-dependent targeting of DLBCL cells. Based on their size, EVs are arbitrarily categorized into multivesicular body-derived small (30–100 nm) or large exosomes (80–120 nm), plasma membrane-derived microvesicles (100–1,000 nm) and apoptotic bodies (>1,000 nm), the latter derived from dying cells. Except for apoptotic bodies, healthy cells constitutively and especially upon stimulation release a mixture of different vesicle types. Notably, EVs can be isolated by different methods and the selected procedure might influence the enrichment of individual types of vesicles. To avoid this risk, we pooled the EV fractions. Another interesting aspect is the fact that EVs show a strong expression of ADAM10, a major sheddase of CD30. The sheddase is not only active on cells but also cleaving CD30 on EVs (Hansen et al., 2016). A slow depletion of CD30 might be the reason that CD30^+^ EVs are barely found in the peripheral blood of cHL patients. The limitation of CD30^+^ EVs to the tumor microenvironment is at least partially explaining the robust clinical efficacy and the mild systemic off-target effects of BV.

## Conclusion

The detailed mode of action of the CD30 antibody-drug conjugate BV in DLBCL is not well understood in all aspects since the clinical outcome seems to be partially independent of the CD30 expression on the tumor cells. Since CD30^+^ bystander cells are enriched in the tumor tissue in many cases of DLBCL, CD30 might however be released with EVs. We thus propose a model that even in the absence of CD30 on the tumor cells to be targeted, EVs can transport the targeting protein from cells of the tumor microenvironment to tumor cells (Figure 4). This *in vitro* model explains the clinical efficacy of BV also in cases when tumor cells lack the targeting antigen. Because CD30 is not only overexpressed on certain lymphomas but also on certain activated bystander cells in other diseases, BV might be a promising therapeutic option to treat other malignancies or immune diseases.

## Data Availability Statement

The datasets presented in this study can be found in online repositories. The names of the repository/repositories and accession number(s) can be found below: The mass spectrometry proteomics data have been deposited to the ProteomeXchange Consortium via the PRIDE partner repository with the dataset identifier PXD025442.

## Ethics Statement

The studies involving human participants were reviewed and approved by Geschäftsstelle Ethikkommision, Universität zu Köln, 50931 Köln. The patients/participants provided their written informed consent to participate in this study.

## Author Contributions

LL was the main investigator who performed most experiments. ML and OJ performed the Image Stream analysis. AS performed the microscopy. FB performed and analyzed the TEM. HD performed and analyzed the DLBCL microscopy. BP and AP performed and evaluated the mass spectrometry. P-HN and MH provided important clinical impact and critically evaluated the manuscript. HH designed and supervised the project. HH, OJ, and AS wrote the manuscript. All authors read and approved the final version of the manuscript.

## Conflict of Interest

HD was employed by the company Pathodiagnostik Berlin MVZ GmbH. The remaining authors declare that the research was conducted in the absence of any commercial or financial relationships that could be construed as a potential conflict of interest.

## Publisher’s Note

All claims expressed in this article are solely those of the authors and do not necessarily represent those of their affiliated organizations, or those of the publisher, the editors and the reviewers. Any product that may be evaluated in this article, or claim that may be made by its manufacturer, is not guaranteed or endorsed by the publisher.

## Supplementary Material

Supplementary Figure 1 Enrichment analysis of the protein hits of the 10,000 x*g* and 100,000 x*g* fraction of L540 EVs.

Supplementary Figure 2 Viability assay of P30-OH/KUBO.

Supplementary Table 1 Gene list of mass spectrum hits of the 10,000 x*g* and the 100,000 x*g* fraction of L540 EVs.
