# Supplementary material for: CD30-Positive Extracellular Vesicles Enable the Targeting of CD30-Negative DLBCL Cells by the CD30 Antibody-Drug Conjugate Brentuximab Vedotin
Source: Front Cell Dev Biol. 2021 Jul 30;9:698503. doi: 10.3389/fcell.2021.698503 (PMC8362802; doi:10.3389/fcell.2021.698503)
Supplement: Supplementary Figure 1 — Enrichment analysis of the protein hits of the 10,000 xg and 100,000 xg fraction of L540 EVs. [file Image_1.pdf]

Supplemental Figure 1

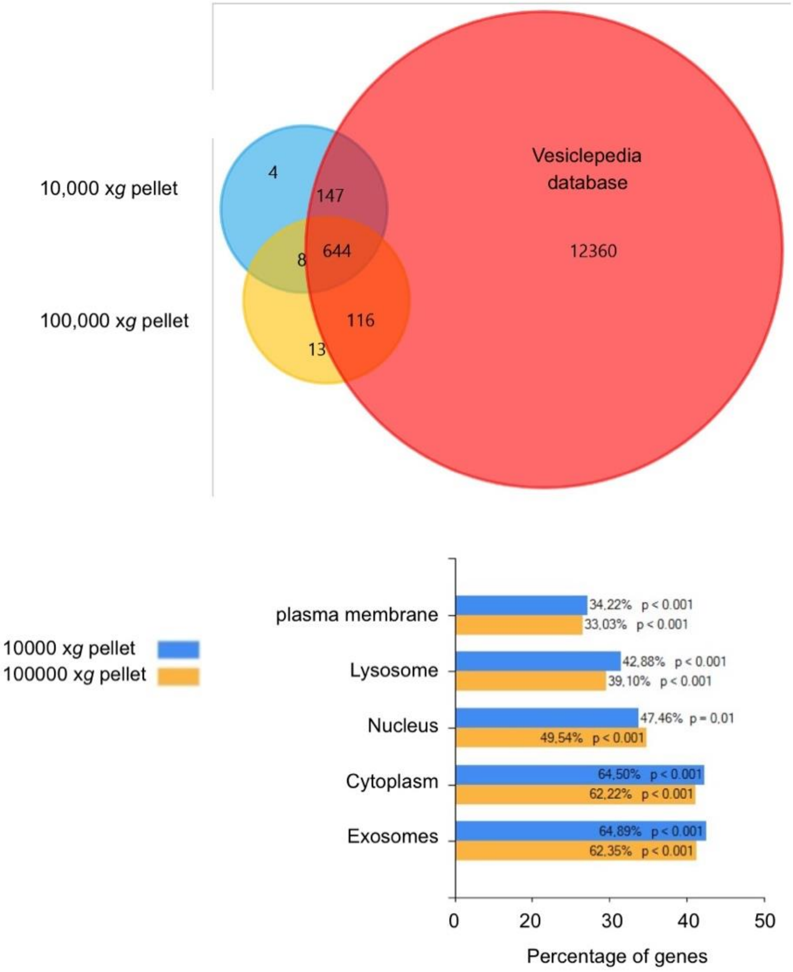

Supplemental Figure 1. Proteomics of the 10,000 x*g* and 100,000 x*g* EV fractions from L540 cells. Mass spectrometry of the EV fractions revealed that they contained a similar amount of protein hits with largely overlapping genes (10,000 x*g* in blue; 100,000 x*g* in yellow). Most of the hits were found in the Vesiclepedia database, i.e. 98.5% and 97.3% of the 10,000 x*g* and 100,000 x*g* fraction respectively. This finding indicates that both fractions contain typical EV proteins. Also, categorization of the protein hits with the Funrich 3.1.4 software, revealed that similar percentages were allocated to different cellular components, whereof the category of exosomes was the most prominent one.

Material and Methods

Identification of EV proteins by MS/MS:

Vesicle proteins were separated by SDS-PAGE (10%). Then, the lanes were excised from the gel, reduced (5 mM dithiotreitol, 25 min at 56°C), alkylated (14 mM iodoacetamide, 30 min at room temperature in the dark), and digested with trypsin (Promega). The resulting peptide mixture was analyzed using an LTQ Velos Orbitrap mass spectrometer (Thermo Fisher Scientific) coupled with LC-MS/MS by an EASY-nLC system (Proxeon Biosystem) through a Proxeon nanoelectrospray ion source. Peptides were separated by a 2-90% acetonitrile gradient in 0.1% formic acid using an analytical column PicoFrit Column (20 cm x ID75 µm, 5 µm particle size, New objective) at a flow rate of 300 nL/min over 45 min. The full scan MS spectra (m/z 300-1,600) were acquired in the Orbitrap analyzer after accumulation to a

target value of $1e^6$. Resolution in the Orbitrap was set to r= 60,000 and the 20 most intense peptide ions with charge states ≥ 2 were sequentially isolated to a target value of 5,000 and fragmented in the linear ion trap by low-energy CID (normalized collision energy of 35%). The signal threshold for triggering an MS/MS event was set to 1,000 counts. Dynamic exclusion was enabled with an exclusion size list of 500, exclusion duration of 60 s, and repeat count of 1. An activation q=0.25 and activation time of 10 ms were used.

Peak lists (msf) were generated from the raw data files using Proteome Discoverer version 1.4 (Thermo Fisher Scientific) with the Sequest search engine and searched against Human Uniprot (91974 sequences; 36693332 residues; released in March 2016) with carbamidomethylation (+57.021 Da) as the fixed modification, oxidation of methionine (+15.995 Da) as the variable modification, one trypsin missed cleavages, and a tolerance of 10 ppm for precursor and 1 Da for fragment ions. The resulting searched data were further analyzed in the Scaffold Q+ software 1.4.0.288. The scoring parameters (Xcorr and protein/peptide probability) were set to obtain a false discovery rate of about 1%. The identified proteins are shown in Supplemental Table 1.
