# Supplementary material for: CD30-Positive Extracellular Vesicles Enable the Targeting of CD30-Negative DLBCL Cells by the CD30 Antibody-Drug Conjugate Brentuximab Vedotin
Source: Front Cell Dev Biol. 2021 Jul 30;9:698503. doi: 10.3389/fcell.2021.698503 (PMC8362802; doi:10.3389/fcell.2021.698503)
Supplement: Supplementary Figure 2 — Viability assay of P30-OH/KUBO. [file Image_2.pdf]

Supplemental Figure 2

Viability assay of P30-OH/KUBO. We determined the functionality of $CD30^+$ EVs regarding the cytotoxicity of BV in $CD30^+$ P30-OH/KUBO. Although cells themselves express CD30, $CD30^+$ EVs were able to support the toxicity of BV. Using 10µg/mL BV EVs significantly enhanced the cytotoxicity of BV.

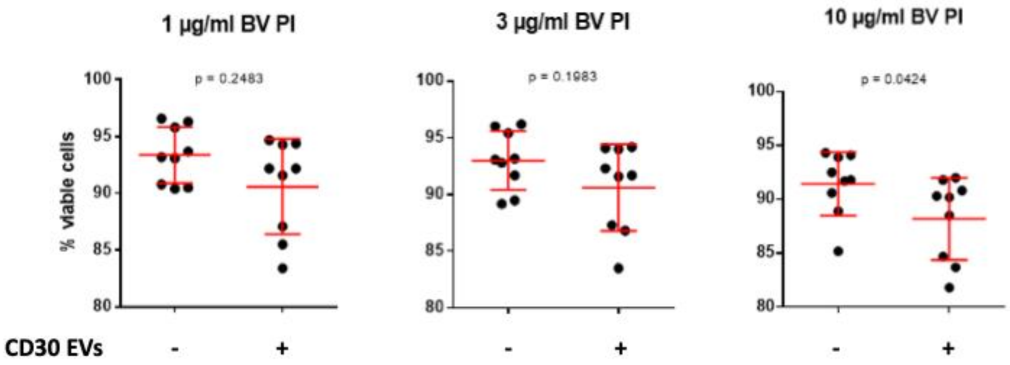

Viability assay of P30-OH/KUBO. Viability assay of P30-OH/KUBO ± EVs from L540 cells with the addition of BV in different concentrations 1, 3 and 10 µg/mL (from left to right). Cells were cultivated for 72 h at 37 °C under standard conditions. The percentage of damaged cells was analyzed by flow cytometry using the propidium iodide (PI) staining. For the statistical analysis the Mann-Whitney test was used.
